# The projection score - an evaluation criterion for variable subset selection in PCA visualization

**DOI:** 10.1186/1471-2105-12-307

**Published:** 2011-07-28

**Authors:** Magnus Fontes, Charlotte Soneson

**Affiliations:** 1 Centre for Mathematical Sciences, Lund University, Box 118, SE-221 00 Lund, Sweden

## Abstract

**Background:**

In many scientific domains, it is becoming increasingly common to collect high-dimensional data sets, often with an exploratory aim, to generate new and relevant hypotheses. The exploratory perspective often makes statistically guided visualization methods, such as Principal Component Analysis (PCA), the methods of choice. However, the clarity of the obtained visualizations, and thereby the potential to use them to formulate relevant hypotheses, may be confounded by the presence of the many non-informative variables. For microarray data, more easily interpretable visualizations are often obtained by filtering the variable set, for example by removing the variables with the smallest variances or by only including the variables most highly related to a specific response. The resulting visualization may depend heavily on the inclusion criterion, that is, effectively the number of retained variables. To our knowledge, there exists no objective method for determining the optimal inclusion criterion in the context of visualization.

**Results:**

We present the projection score, which is a straightforward, intuitively appealing measure of the informativeness of a variable subset with respect to PCA visualization. This measure can be universally applied to find suitable inclusion criteria for any type of variable filtering. We apply the presented measure to find optimal variable subsets for different filtering methods in both microarray data sets and synthetic data sets. We note also that the projection score can be applied in general contexts, to compare the informativeness of any variable subsets with respect to visualization by PCA.

**Conclusions:**

We conclude that the projection score provides an easily interpretable and universally applicable measure of the informativeness of a variable subset with respect to visualization by PCA, that can be used to systematically find the most interpretable PCA visualization in practical exploratory analysis.

## Background

High-dimensional data sets, where the observed variables often by far outnumber the samples, are becoming increasingly prevalent in many scientific domains. As an example, microarrays are used extensively to measure a variety of genomic attributes such as gene expression and DNA copy numbers. A peculiar feature of many high-dimensional data sets is that they are often collected with an exploratory aim, without a specific hypothesis in mind. This means, among other things, that a data set may contain many variables that are not really informative, while the informative structure is contained in a small subset of the variables. The presence of non-informative variables can have detrimental effects on the possibility to extract relevant biological knowledge from the observed data, and the performance of many machine learning algorithms may drastically improve if the number of variables is reduced before or in conjunction with the application of the algorithm.

The exploratory perspective on high-dimensional data means that visualization methods, providing a graphical representation of the data, can be of great assistance. One of the most commonly used visualization methods for microarray data is Principal Component Analysis (PCA) [1-4] which provides coupled, low-dimensional representations of the samples and variables in a data set. PCA is applicable also to very high-dimensional data sets, but the resulting visualization can be severely confounded and the truly informative structure can be concealed by the presence of a large number of non-informative variables. Hence, variable selection can be of great importance to improve the interpretability of PCA visualizations. In the microarray context, one commonly used approach to reduce the dimensionality is to simply exclude the variables with the lowest variances before applying PCA [5-8], implicitly assuming that they do not contain any useful information concerning the samples in the data set. Such variable filtering often provides more informative and easily interpretable PCA representations. Other ways to reduce the dimension before PCA are also common, such as excluding the variables with the lowest signal intensities [9] or including only those variables that are highly related to a specific response [6,7,10].

Naturally, the resulting visualization may be highly dependent on the number of included variables and, hence, on the variance or significance threshold used as the inclusion criterion. To our knowledge, there exist no well-motivated, objective criteria that are useful for obtaining good stopping rules in such variable filterings, and therefore most studies apply some kind of ad-hoc criterion. In this paper, we present the *projection score*, which is a straightforward, intuitively appealing measure of the informativeness of a variable subset with respect to visualization. Intuitively, the projection score compares the variance captured by a pre-defined set of principal components to the expected value under the assumption that the variables are independent, that is, that there is no non-random structure in the data. The projection score can be universally applied to provide a stopping rule for a variable filtering, and hence it can be used to systematically find the most interpretable PCA visualizations in practical exploratory analysis applications, for example in microarray data analysis. Moreover, as we will see in this paper, the projection score can be applied in more general contexts than those indicated above, to compare the informativeness of any variable subsets with respect to visualization by PCA.

## Related work

Many variable selection techniques have been devised for use with supervised learning algorithms, such as classifiers or predictors. In the supervised learning context, measuring the informativeness of a variable subset is fairly straightforward. Probably the most common approach is to use some kind of cross-validation scheme to train the classifier on the samples in a training set and define the informativeness of the variable subset based on the performance of the obtained classifier in an independent test set [11]. For unsupervised learning methods, such as visualization and clustering, which do not make use of any class labels or other response variables, this approach is not applicable. Variable selection for model-based clustering, where the data are assumed to come from a mixture of several subpopulations, has been considered [12,13]. In this case, the value of the objective function depends on how well the data comply with the assumed mixture model. For visualization purposes however, it is less clear what constitutes an optimal result. The projection score defined in this paper measures the informativeness of a variable subset based on the variance accounted for by a subset of the principal components of the variable subset, without using any side information such as the class memberships of samples.

Variable selection in the context of PCA has been considered for many years, but often with the aim of approximating the original data set as well as possible with only a subset of the variables. Moreover, the proposed methods do not in general address the problem of finding a suitable stopping criteria for variable filtering. The potential advantage of sparse components, in terms of interpretability, were recognized already in the 1970s [14,15]. One common approach to variable selection for PCA has been to fix the desired number of variables in advance and search for the optimal variable subset of this pre-selected cardinality [4,16-19]. To comply with the original goals of PCA, the optimal variable subset is often defined as the one containing the largest amount of variance, or providing the best approximation to the original data set. Hence, one searches for a subset of the variables containing essentially the same information as the entire variable set. This goal is common also to many of the recently proposed sparse PCA methods [20-22]. These methods often take their starting point in one of the optimality properties of ordinary PCA and introduce a penalty (often a lasso [23] or elastic net [24] penalty) to limit the number of non-zero principal component weights. The algorithms are in general developed for a fixed penalty (that is, a fixed number of non-zero weights) and the optimal penalty is determined by cross-validation, trying to approximate a test set as well as possible, or by ad-hoc methods. In contrast to these methods, the main objective of the approach proposed in this paper is to compare subsets of *different *sizes, such as, for example, subsets obtained by variance filtering with different inclusion thresholds. Our aim is not explicitly to approximate the original data set as well as possible, but rather to find informative structures which may not be apparent by considering the entire data set. By filtering the variable set with respect to a specific factor (such as variance or the association with a response) we obtain sparse principal components where the included variables are all related to the same factor. These may be easier to interpret than general sparse principal components. With our approach, it is also possible to compare the informativeness contained in different factors, by filtering with respect to the association with each one of them and comparing the best projection scores obtained for each factor.

Another related approach for variable selection, or variable clustering, using PCA is the gene shaving procedure [25,26]. This procedure starts by computing the first principal component of the data. In each step, the variables with the smallest loadings are shaved off, and a new principal component is computed from the remaining variables. This yields a nested sequence of variable subsets. For each subset, a quality measure is computed as the ratio between the variance of the mean value of the expression levels of the genes in the subset and the total variance of the subset. Then, this quality measure is compared to what would be expected from random data, using the gap statistic (that is, the difference between the observed and the expected value). The variable subset with the highest value of the gap statistic is considered to be the optimal variable subset (called a gene cluster). We will show that one way to use the projection score is to obtain another quality measure of the variable subsets that can be used in such a shaving approach, and that the optimal variable set is not necessarily the same as with the method described in [25,26].

Biclustering methods based on sparse matrix decompositions have been proposed by several authors (see for example [27] and references therein). Here, the aim is to find a subset of the variables which are correlated across a subset of the samples and hence sparsity is induced for both samples and variables. The biclustering methods use different measures to evaluate subsets of a given data set. For example, in [28] the quality of a submatrix is defined based on the average expression value in the submatrix. Biclustering methods have been shown to be useful for finding informative patterns which are not necessarily present across the entire data set. However, they are not explicitly optimized for visualization, and, as the sparse PCA methods, they are not used to find stopping criteria for variable filtering.

Another variable selection criterion for PCA was described in [29]. For each variable, the authors compute the difference between the entropy of the entire data set and the entropy of the data set with the variable removed. This is used as a measure of the contribution of each of the variables, and the variables are ranked according to their contributions. The optimal variable subset is considered to consist of all variables whose contributions are more than one standard deviation higher than the mean value of all contributions. In contrast to this method, we compute the projection score for a large number of variable subsets and select the one with the highest value as the most informative in a visualization context.

## Results and Discussion

In this section, we will first apply different filtering techniques, with varying inclusion criteria, to generate a collection of variable subsets from each of three microarray data sets. The projection score will be applied to find the most informative variable subset in each example. It is important to note that the informativeness is measured with respect to unsupervised, exploratory analysis by PCA, where the aim often is generation of new hypotheses rather than validation of existing hypotheses. This means that we could use the projection score as a quality measure even in the absence of any side information about, for example, sample groups. It can also be used to quantify and compare the informativeness of variable subsets obtained by supervised methods, for example subsets consisting of the variables most highly related to a given response. Regardless of how the variable subsets are obtained, the projection score evaluates their informativeness from an unsupervised perspective, based on the variance contained in a pre-defined subset of the principal components of the respective variable subsets.

In all cases, when we vary the inclusion criterion (in most of our examples, a single parameter), the projection score traces out a reasonably smooth curve, often with a clear maximum, which means that it is reasonable to say that there is indeed a maximally informative subset in the proposed sense. In this article, this curve will be referred to as the *projection score curve *for the filtering parameter. In the first example, we filter the variable set by variance and find an informative subset of variables with high variances, providing a graphical sample representation which is more easily interpretable than the one obtained from all variables. In the second example, we filter the variable set by the association with given responses, and show that the optimal projection score and the shape of the projection score curve obtained from filtering with respect to a certain response capture the overall evidence in the data for a significant association with that response. We also show the results from a combined variance and response-related filtering. In the third example, we apply a shaving procedure to generate variable subsets, and evaluate the obtained subsets by their projection scores.

Then, we validate the use of the projection score with synthetic examples, where we show that the variable subset with the highest projection score is often the one containing the non-random structure in the data. Finally, we discuss some issues regarding the estimation of the projection score and warn against a potential pitfall.

In all examples, we use the projection score to compare the informativeness of variable subsets with respect to visualization. Assume that we are given a data set with *N *samples and *p *variables, represented by a rank-*r *matrix **X **∈ ℝ^*p*×*N*^, and a collection of *M *variable subsets *R_m _*⊆ {1, ..., *p*} for *m *= 1, ..., *M*. We define selection functions *ϕ_m _*in such a way that  consists only of the rows in **X **with indices in *R_m_*. To compute the projection score, we must also select an *S *⊆ {1, ..., *r*}, essentially representing the number of degrees of freedom we expect in the data. Letting **Λ_X _**= (*λ*_1_, ..., *λ_r_*) denote the vector of non-zero singular values of **X **in decreasing order, the fraction of the variance in **X **which is accounted for by the principal components with indices in *S *can be computed as

The projection score of *R_m _*is then defined as

Here,  denotes the inferred distribution of *ϕ_m_*(**X**) under the assumption of independence among the original samples and variables in **X**. We compute the projection score for each of the *M *variable subsets, and the subset with the largest projection score is considered the most informative variable subset for visualization. For further details, see the Methods section. To obtain a visualization which reflects the correlation structure between the variables of the data set instead of the individual variances, we consistently extract the singular values from standardized data matrices, where each variable is mean-centered and scaled to unit variance. This standardization is indeed commonly used [30,31]. If we had not standardized the data, the correlations between the variables would be less influential when computing the principal components, and the variances of the individual variables could potentially have a very large impact. Since we define the projection score by comparing the observed data to data generated under a null model defined by assuming independence between the variables, we may argue that the non-random structure that is detected with the resulting score is that corresponding to correlations between variables and that therefore, the standardized data are better suited for our purposes. Note that for the variance- and response-related filterings, the variable rankings are extracted from the original, unstandardized data.

### Variance filtering of a lung cancer data set

**Figure 1:**
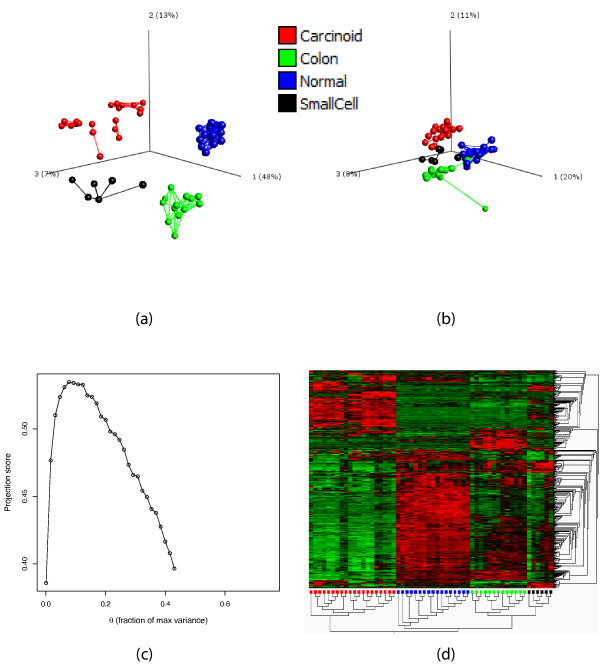
**Variance filtering of the lung cancer data**. (a) The sample representation obtained by applying PCA to the most informative variable subset obtained by variance filtering, containing 591 genes. The different colors indicate different cancer subtypes. (b) The sample representation obtained by applying PCA to the entire variable set (12,625 variables). (c) The projection score as a function of the variance threshold *θ *(fraction of maximal variance) used for inclusion. (d) Heatmap of the most informative variable subset, that is, the one used to create the sample representation in (a). In panels (a) and (b), in order to obtain more easily interpretable plots, we joined the closest neighbors among the samples with line segments. The distance between two samples is defined by the Euclidean distance in the space spanned by all the remaining variables. The hierarchical clusterings in panel (d) are created using Euclidean distances and average linkage. The figures in (a), (b) and (d) were generated using Qlucore Omics Explorer 2.2 (Qlucore AB, Lund, Sweden).

We first use the projection score to find particularly informative variable subsets among those obtained by applying variance filters to the lung cancer data set studied in [27,32]. The data set contains gene expression measurements for 12,625 genes in 56 subjects. The subjects belong to four groups: Normal (*N *= 17), Pulmonary carcinoid tumors (*N *= 20), Small cell carcinoma (*N *= 6) or Colon metastases (*N *= 13). For a given set of variance thresholds  (defined for the original, unstandardized data), we define *R_m _*as the collection of variables with variance exceeding *θ_m_*. The variance thresholds are specified as fractions of the highest individual variable variance in the data set, meaning that the variables included in the variable subset *R_m _*are those with variances exceeding *θ_m _*· *var*_max _where *var*_max _denotes the maximal variance among the variables in the data set. We choose *S *= {1, 2, 3}, that is, we search for a variable subset providing an informative three-dimensional sample representation, which is reasonable in a visualization context. Figure 1(a) shows the three-dimensional sample representation obtained by applying PCA only to the variables in the most informative subset. The representation based on the most informative subset is considerably more easily interpretable than the representation based on the entire set of variables, which is shown in Figure 1(b). In the representation based on the most informative variable subset, the pulmonary carcinoid tumors (shown in red) appear to cluster into several subgroups, an effect which was also noted in [27]. Figure 1(c) shows the projection score curve for the filtering parameter *θ*. Very small variable subsets, corresponding to high values of the variance threshold, do not support the chosen *S *(see Methods section). The projection score attains its maximal value of *τ *= 0.534 for a variance threshold of 7.76% of the maximal variance, corresponding to a variable subset consisting of the 591 variables with highest variances. In Figure 1(d), we show a heatmap for the expression matrix corresponding to the most informative variable subset. Clearly, the variables with the highest variances contain much information about the four sample groups, which is not surprising.

### Response-related filtering of the NCI-60 data set

In this section, we construct *ϕ_m_*(**X**) to consist of the variables which are most highly related to a given response variable. In the studied examples the response variable indicates the partition of the samples into different groups. Given such a partition, we calculate the *F*-statistic, contrasting all these groups, for each variable. For a given set of significance thresholds , all variables which are significantly related to the response at the level *α_m _*(that is, all variables with a *p*-value below *α_m_*) are included in *ϕ_m_*(**X**). For each randomized data set X* used to estimate  (see the Methods section) we define the significance thresholds  in such a way that the resulting variable subsets have the same cardinalities as the corresponding subsets from the original data set. The choice of *S *is guided by the underlying test statistic. When we contrast only two groups, we do not expect the optimal variable subset to support more than a one-dimensional sample configuration, and therefore we choose *S *= {1} in this case. When contrasting more than two groups, the choice of *S *must be guided by other criteria. This is because the variables with the highest *F*-score may in this case very well characterize many different sample groups, not all of which can simultaneously be accurately visualized in low dimension.

**Figure 2:**
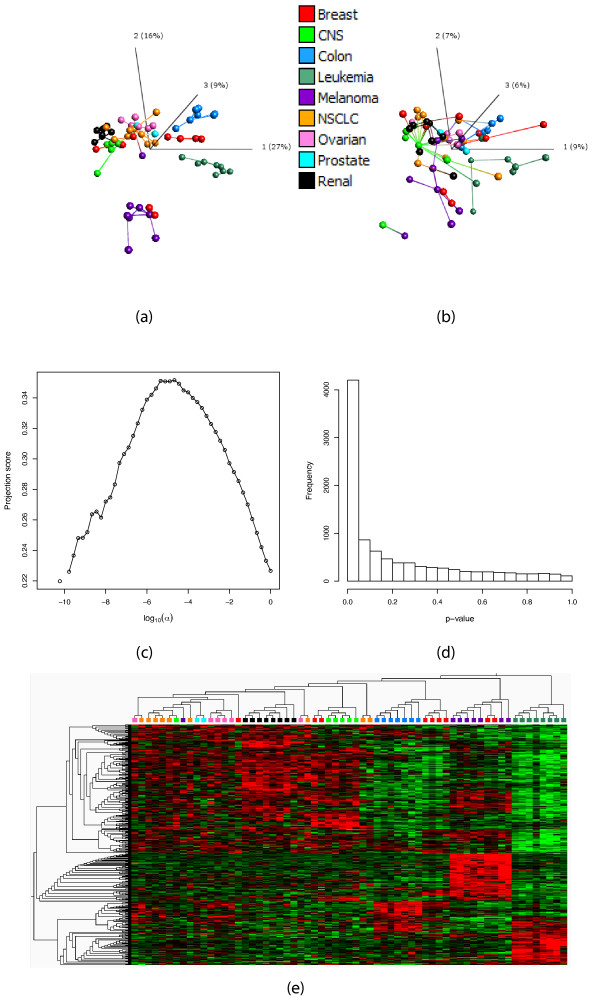
**Response-related filtering of the NCI-60 data**. (a) The sample representation obtained by applying PCA to the most informative variable subset (482 variables) obtained by filtering with respect to the F-value for contrasting all nine cancer types. The different colors indicate different cancer types. (b) The sample representation obtained by applying PCA to the entire variable set (9,706 variables). (c) The projection score as a function of log_10_(*α*), where *α *is the *p*-value threshold used for inclusion. (d) The *p*-value distribution for all variables, indicating that there are truly significantly differentially expressed genes with respect to the contrast. (e) Heatmap of the most informative variable subset, that is, the one used to create the sample representation in (a). In panels (a) and (b), in order to obtain more easily interpretable plots, we joined the closest neighbors among the samples with line segments. The distance between two samples is defined by the Euclidean distance in the space spanned by all the remaining variables. The hierarchical clusterings in panel (e) are created using Euclidean distances and average linkage. The figures in (a), (b) and (e) were generated using Qlucore Omics Explorer 2.2 (Qlucore AB, Lund, Sweden).

The NCI-60 data set [33] contains expression measurements of 9,706 genes in 63 cell lines from nine different types of cancers. We first filter the variable set with respect to the association with the partition defined by all the nine cancer types, using *S *= {1, 2, 3}. This gives a most informative subset consisting of 482 variables, with a projection score *τ *= 0.351. The resulting sample representation, obtained by applying PCA to the most informative variable subset, is shown in Figure 2(a). The representation based on all variables is shown in Figure 2(b) and the projection score is shown in Figure 2(c) as a function of log_10_(*α*). Figure 2(d) shows the *p*-value distribution, which indicates that there are indeed variables which are truly significantly associated to the response. Figure 2(e) shows a heatmap for the most informative variable subset (the same as in Figure 2(a)) where it can be seen that the samples are reasonably well clustered according to cancer type based on these 482 variables only.

**Figure 3:**
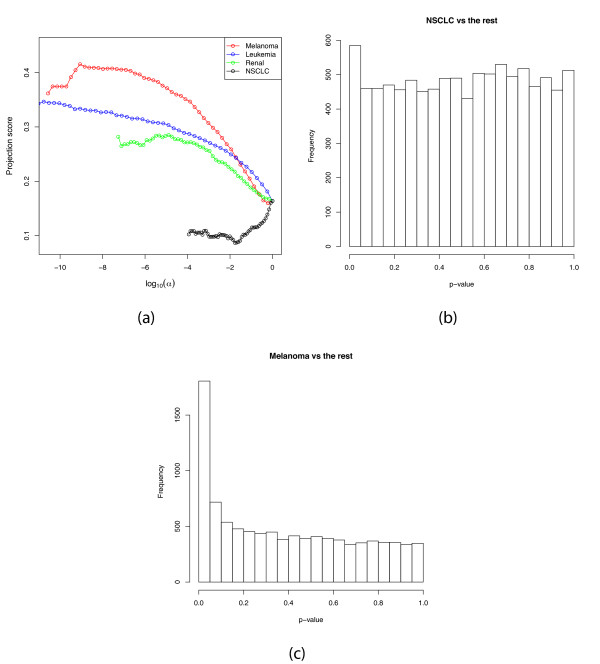
**Response-related filtering of the NCI-60 data - continued**. (a) The projection score as a function of log_10_(*α*), where *α *is the *p*-value threshold used for inclusion when contrasting each of four cancer types with all the other eight types in the NCI60 data set. For the Melanoma, Leukemia and Renal types, small groups of variables form the most informative subsets. For the NSCLC type, the entire variable collection is the most informative variable subset. (b) The *p*-value distribution for all variables when contrasting NSCLC with all other groups, indicating that there are essentially no truly significantly differentially expressed genes for this contrast. (c) The *p*-value distribution for all variables when contrasting Melanoma with all other groups.

Next, we set out to study how informative the contrasts between one cancer type and all the others are. We filter the variable set using the association with the contrast of one cancer type vs the rest, using *S *= {1}. Figure 3(a) shows some of the projection score curves. First, we can note that the range of *p*-values, as well as the range of obtained projection scores, are highly different for the different contrasts. The highest projection scores in the respective cases are 0.416 (for the Melanoma vs the rest contrast), 0.348 (Leukemia), 0.281 (Renal) and 0.164 (NSCLC). Apparently, for each of the Melanoma, Leukemia and Renal contrasts, a small subset of the variables related to the respective response contains a lot of non-random information. However, for the NSCLC contrast the full variable set (corresponding to log_10_*α *= 0) is the most informative. This can be understood from Figure 3(b), which shows a histogram over the *p*-values obtained from the *F*-test contrasting the NSCLC group with the rest of the samples. The *p*-values are essentially uniformly distributed, indicating that there are no truly differentially expressed genes in this case. Hence, in the filtering process we do not unravel any non-random structure, but only remove the variables which are informative in other respects. Figure 3(c) shows the *p*-value distribution for the Melanoma contrast. In this case, there are indeed some differentially expressed genes, which means that in the filtering process, we purify this signal and are left with an informative set of variables. The projection scores obtained from the different contrasts are consistent with Figure 2(a), in the sense that the highest projection scores are obtained from the contrasts corresponding to the cancer types which form the most apparent clusters in this sample representation, that is, the Melanoma samples and the Leukemia samples. The NSCLC samples do not form a tight cluster and are not particularly deviating from the rest of the samples in Figure 2(a).

### Combined variance and response-related filtering of the NCI-60 data set

**Figure 4:**
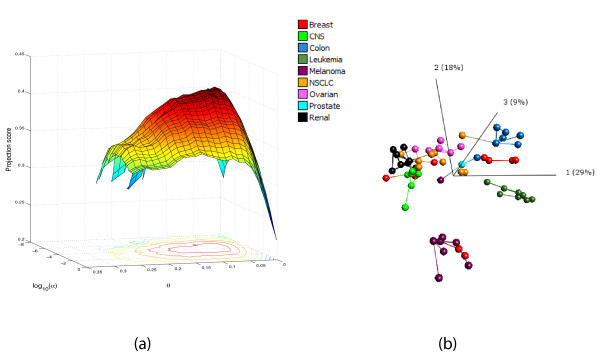
**Combined variance and response-related filtering of the NCI-60 data**. (a) The projection score for various choices of the inclusion criteria for the variance (*θ*, fraction of max variance) and the *p*-value from an *F*-test contrasting all nine cancer types (log_10_(*α*), where *α *is the *p*-value threshold). The optimal projection score is obtained by combining the two filtering procedures. (b) The sample representation obtained by applying PCA to the most informative variable subset. In panel (b), in order to obtain a more easily interpretable plot, we joined the closest neighbors among the samples with line segments. The distance between two samples is defined by the Euclidean distance in the space spanned by all the remaining variables. The figure in (b) was generated using Qlucore Omics Explorer 2.2 (Qlucore AB, Lund, Sweden).

In some cases, the variable set is first filtered by variance before a statistical test is applied. Removing supposedly non-informative variables in this way may be beneficial in terms of statistical power, since if the number of performed tests is high, we need to do a rather heavy correction for multiple comparisons. Also with the approach proposed in this paper, we can combine different filtering procedures to find a variable subset which is informative from more than one perspective. Here, we show how to combine variance filtering with response-related filtering for the NCI-60 data set. In this way, we exclude all variables that obtain small *p*-values from the *F*-test mostly due to their low variances. As before, we define a collection of variance thresholds *θ_m _*and a number of significance thresholds *α_m_*, for the *F*-test contrasting all nine cancer types. We choose *S *= {1, 2, 3} as before. Now, the projection score can be represented as a surface, shown in Figure 4(a), for varying values of the variance and *p*-value thresholds. The curves traced out for log_10_(*α*) = 0 and *θ *= 0 correspond to the projection score curves for the individual variance and response-related filterings, respectively. As can be seen in Figure 4(a), we get the maximal projection score for a variable subset obtained by a combination of variance filtering and response-related filtering. This subset includes all variables with a variance exceeding 5.8% of the maximal variance, and with a *p*-value below 3.6 · 10^-5^, in total 263 variables. The maximal projection score is *τ *= 0.416. This can be compared to the purely response-related filtering, which gave a maximal projection score of *τ *= 0.351 by including the 482 variables with *p*-values below 2.1 · 10^-5^. Figure 4(b) shows the sample configuration obtained by applying PCA to the most informative variable subset from the combined filtering.

### Shaving of a leukemia data set

Here, we will use the projection score to evaluate the informativeness of variable subsets obtained by the shaving procedure described in [25]. Briefly, for a given fraction *π *∈ (0, 1), we define a function *ξ *: 2^{1,...,*p*} ^→ 2^{1,...,*p*} ^by letting *ξ*(*R*) consist of the fraction 1 - *π *of the variables in *R *having the highest loadings in the first principal component of the data set consisting of the variables in *R*. The selection functions *ϕ_m _*are then defined by letting *ϕ_m_*(**X**) contain all variables in .

We use *π *= 0.02, hence in each step shaving off 2% of the variables, continuing until only one variable remains. We compute the projection score for each of the variable subsets, as well as the gap statistic proposed in [25] (using the signed variables, see Methods section).

**Figure 5:**
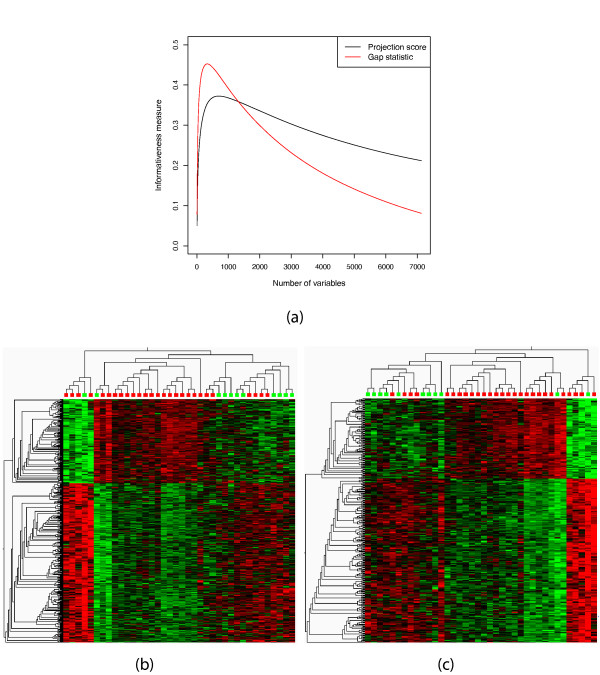
**Gene shaving of the leukemia data**. (a) The projection score (black) and the gene shaving gap statistic (red) as functions of the cardinality of the variable subset. (b) Heatmap of the most informative variable subset by the projection score, consisting of 691 variables. Red - ALL, green - AML. (c) Heatmap of the most informative variable subset by the gene shaving gap statistic, consisting of 336 variables. The hierarchical clusterings in panels (b) and (c) are created using Euclidean distances and average linkage. The figures in (b) and (c) were generated using Qlucore Omics Explorer 2.2 (Qlucore AB, Lund, Sweden).

We apply the described method to the leukemia data set studied in [34]. The data set contains gene expression measurements from 7,129 genes in 38 samples with either AML (*N *= 11) or ALL (*N *= 27). Computing the projection score and the gap statistic for the variable subsets obtained by shaving gives optimal variable subsets containing 691 variables (projection score) and 336 variables (gap statistic), respectively. Figure 5(a) shows the two informativeness measures as functions of the variable subset cardinality. Both curves are smooth and have clear extreme points. Figures 5(b) and 5(c) show heatmaps for the optimal variable subsets from the two methods. As can be seen in these figures, the two subsets contain much the same information about the samples in the data set. The variable subset selected by the projection score is larger than the one selected by the gap statistic. In some situations, a small number of included variables may be beneficial. However, as will be shown (Tables 1 and 2, see the section "Detecting sparsity in principal components" below), the gap statistic tends to underestimate the number of variables in the optimal subset (this was also noted in [26]), which may potentially be the case also in this example. This would then lead to a number of "false negatives", that is, variables falsely excluded from the optimal combination.

**Table 1:** Sparsity detection, example 1.

Measure	First component	Second component
Projection score	10 (10-10)	10 (9-10)
Gap statistic	10 (9-10)	10 (8-10)
SPC	18.5 (10-46)	21 (11-32)
SSVD	11 (10-13)	10.5 (1-12)

The median and range (across 10 instances) of the number of non-zero elements included in the optimal variable subset found by different methods. Each underlying component contains 10 non-zero elements. SPC - sparse PCA [22]. SSVD - sparse SVD [27]. The gap statistic is defined as in [25,26].

**Table 2:** Sparsity detection, example 2.

Measure	First component	Second component
Projection score	160 (156-160)	152 (133-156)
Gap statistic	122.5 (106-133)	78 (60-100)
SPC	260.5 (229-499)	311.5 (280-494)
SSVD	162.5 (161-339)	164 (162-313)

The median and range (across 10 instances) of the number of non-zero elements included in the optimal variable subset found by different methods. Each underlying component contains 160 non-zero elements. SPC - sparse PCA [22]. SSVD - sparse SVD [27]. The gap statistic is defined as in [25,26].

### Validation on synthetic data

In this section, we will validate the projection score by using it to find informative variable subsets from different filtering processes applied to synthetic data sets.

#### Variance filtering

We let

and generate a synthetic data set with 1,000 variables and 100 samples by letting

The only non-random structure in the data is contained in the first 150 variables, discriminating between two groups of 50 samples each. By varying *σ*_1 _we obtain data sets with difference variance properties. With *σ*_1 _= 0.5, the informative variables and the non-informative variables have comparable variances. With *σ*_1 _= 0.2, the informative variables obtain a lower variance than the non-informative variables and with *σ*_1 _= 0.8 the informative variables are also those with the highest variances.

We take *S *= {1}, since no other choice of *S *is supported by any variable subset. This is also consistent with the structure of the data matrix.

Across 20 realizations, the mean number of variables in the subset giving the best projection score are 710.2 for *σ*_1 _= 0.5 (standard deviation 143.1), 999.9 for *σ*_1 _= 0.2 (standard deviation 0.30) and 118.3 for *σ*_1 _= 0.8 (standard deviation 15.0). The projection score correctly indicates that when *σ*_1 _= 0.2, the informative structure in the data is indeed related to the variables with lowest variances, and hence all variables are included in the most informative subset (that is, no variance filtering). Note that the association between the variables within each sample group is very strong when *σ*_1 _= 0.2. If the variables with lowest variance had been routinely filtered out in this example, we would lose the informativeness in the data. It can also be noted that when the number of variables is below a certain threshold (approximately 850) in the *σ*_1 _= 0.2 case, not even *S *= {1} is supported by the data since we have filtered out all the informative variables. For *σ*_1 _= 0.5, the optimal number of variables is highly dependent on the specific data set since the filtering removes both informative and non-informative variables uniformly.

#### Response-related filtering

In this example, we simulate a data matrix containing two group structures (see [35]). The data set consists of 40 samples which are divided into four consecutive groups of 10 samples each, denoted *a*, *b*, *c*, *d*. We define

The data matrix is then generated by letting

**Figure 6:**
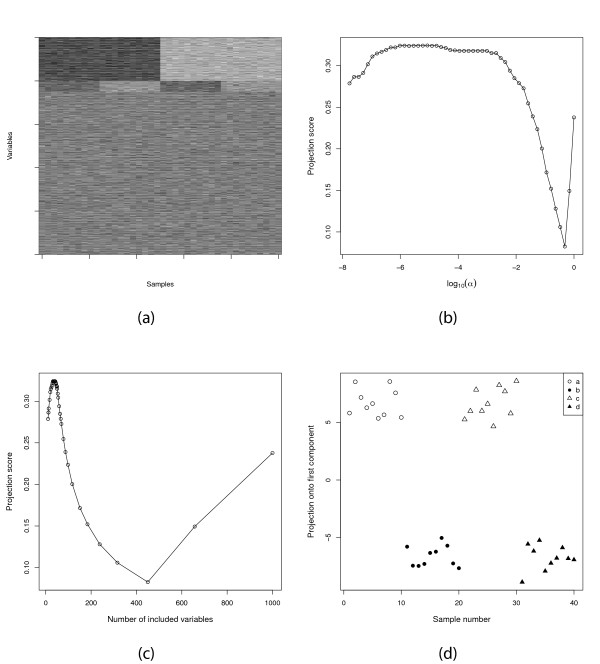
**Response-related filtering of a synthetic data set**. (a) The structure of the data set. The columns represent samples and the rows represent variables. (b) The projection score as a function of log_10_(*α*), where *α *is the *p*-value threshold used as inclusion criterion. (c) The projection score as a function of the cardinality of the variable subset. (d) The projection of the samples onto the first principal component from the most informative variable subset, consisting of 40 variables.

We perform a two-sided *t*-test contrasting *a *∪ *c *and *b *∪ *d *and order the variables by the absolute value of their *t*-statistic for this contrast. In this case, since we compare only two groups we are essentially searching for a one-dimensional separation, so we choose *S *= {1}. Figure 6(a) shows the structure of the data set. The data set contains one very strong factor, encoded by the first 200 variables, and one weaker factor, the one we are interested in, which is related to the next 50 variables. Figures 6(b) and 6(c) show the projection score for different thresholds on the significance level *α *and for different variable subset cardinalities, respectively. The optimal projection score (approximately 0.33) is obtained for variable subsets containing slightly less than 50 variables (mean value across 20 simulations of 38.0, standard deviation 4.6, range 30-46). Hence, even though there is indeed much information contained in the entire variable set, it is possible to obtain an even more informative variable subset by filtering. Projecting onto the first principal component based only on the variables in the most informative subset clearly discriminates between *a *∪ *c *and *b *∪ *d*, as shown in Figure 6(d). As above, we can compare the maximal projection score corresponding to filtering by the association with different responses. Filtering with respect to the association with the contrast between *a *∪ *b *and *c *∪ *d*, that is, the stronger factor in the data set, gives a maximal projection score around 0.60. Hence, this correctly suggests that this factor is more informative than the previously studied. Filtering with respect to the variance, still using *S *= {1}, gives a maximal projection score of 0.68, obtained for approximately 200 variables (the variables related to the first factor in the data). This implies that the variables with high variance in this case are even more informative than the variables closely associated with one of the responses, in the sense that the encoded information deviates more from what would be expected from the highly varying variables in a randomized data set. Applying variance filtering with *S *= {1, 2} provides a most informative variable subset containing 222 variables, capturing parts of both the two informative variable groups in the data (note that the variances of the variables in the second, smaller group are not all larger than the variances of the non-informative variables). *S *= {1, 2, 3} is not supported by any variable subset.

#### Detecting sparsity in principal components

In this example, we will evaluate the usefulness of the projection score for detection of sparsity in the leading principal components of a data matrix. We generate a data set, with *p *= 500 variables and *N *= 50 samples, following the scheme given in [21]. Briefly, we form a matrix **V **= [**v**_1_, ..., **v***_p_*] from an orthonormal set of *p *principal components **v**_1_, ..., **v***_p _*∈ ℝ*^p^*, and a matrix **C **= *diag*(*c*_1_, ..., *c_p_*) containing the eigenvalues in decreasing order on the diagonal. Then, we form the covariance matrix **Σ **by

To generate data, we let **Z **be a random draw from  and take **X **= **VC**^1/2^**Z**. The first two principal components (**v**_1 _and **v**_2_) are constructed to have specific sparsity patterns. In the first example, we let **v**_1 _and **v**_2 _contain 10 non-zero elements each (all of equal magnitude and selected in such a way that the two sets of non-zero elements are non-overlapping) and choose *c*_1 _= 30, *c*_2 _= 16. We let *c_k _*= 1 for *k *= 3, ..., 500 and sample the entries of the corresponding principal components uniformly between 0 and 1 before orthogonalizing and normalizing them. In the second example, we let **v**_1 _and **v**_2 _instead contain 160 non-zero (equal) elements. We choose *c*_1 _= 400 and *c*_2 _= 200 and proceed as in the previous example to obtain the rest of the components.

The variable subsets are obtained by the shaving procedure, as outlined above and described in [25]. This gives a nested sequence of variable subsets, which can be evaluated in terms of their projection scores. We also compute the gap statistic as defined in [25] as another quality measure of each variable subset. The optimal variable subsets obtained from these methods are compared to those found by a sparse PCA algorithm [22] and the sparse SVD proposed in [27]. It should be noted that the aims of these methods are somewhat different. The sparse PCA attempts to find a good approximation of the original data set using only a subset of the variables, while the sparse SVD was developed for biclustering, that is, finding small groups of variables which are related across possibly only a subset of the samples. Hence, also the sample representation can be sparse (that is, only describing some of the samples) in the results from the sparse SVD. In the examples studied here, however, the sparsity patterns of the principal components are designed to be identical across all samples. The sparse SVD and the sparse PCA were applied through the R programs provided by the respective authors. The sparse SVD was applied with *γ_u _*= *γ_v _*= 2, as suggested by the authors. The sparsity parameter in the sparse PCA was estimated via the cross-validation function provided with the R package, evaluating 100 different sparsity levels between 1 and  via 5-fold cross-validation.

Table 1 and Table 2 give the median and the range of the number of non-zero entries in the first two principal components across 10 instances of each example. To obtain the second component, we orthogonalized the observed data matrix with respect to the first component and extracted the first component from the orthogonalized matrix. The results in Table 1 and Table 2 suggest that the projection score is able to detect the underlying sparsity structure of the principal components. The gap statistic tends to underestimate the number of variables in the optimal variable subset, as was also noted in [26]. The sparse PCA consistently overestimates the number of non-zero entries of the components. The sparse SVD performs well in many cases, but the variability is larger than for the projection score-based method.

### Estimating 

**Figure 7:**
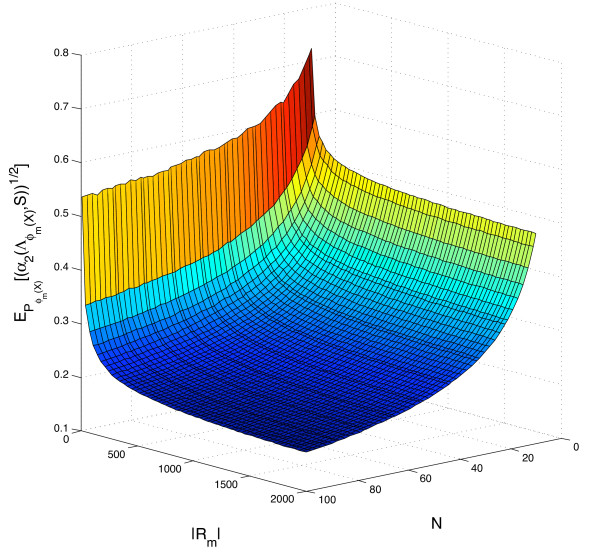
**Estimation of **. The figure shows  with *S *= {1, 2}, for different choices of |*R_m_*| and *N*, when the null distribution  is defined by assuming that the matrix elements are drawn independently from . Red values correspond to high values of , while blue values correspond to low values of this statistic.

To obtain , we repeatedly permute the values in each row of **X **and perform the variable filtering, which is computationally expensive (see Methods section). A more efficient implementation can be obtained if we specify the distribution  for each *m *= 1, ..., *M *in advance. Then, the values of  can be estimated in advance and stored, which leaves only the calculations for the observed data matrix and the subtraction of a known quantity for each variable subset. For instance, we can fix  by assuming that each element in *ϕ_m_*(**X**) is drawn from a standard normal distribution, that is,

for *i *= 1, ..., |*R_m_*| and *j *= 1, ..., *N*. We can then calculate the expected value of  for a large collection of choices of variable subset cardinalities and sample sizes. Figure 7 shows an interpolated surface for 10 ≤ |*R_m_*| ≤ 2, 000 and 10 ≤ *N *≤ 100. This more computationally efficient approach may be used for example for variance filtering, if the observed data matrix is standardized before the principal components are extracted.

When the variable subsets are defined by response-related filtering, it is more difficult to specify and sample from the truncated distribution resulting from the filtering. In particular, we note that even for a data matrix **X **containing only independent variables, we still expect to see an aggregation of the variance in the first principal components when we consider small submatrices *ϕ_m_*(**X**). In some cases, however, it may still be of interest to compare the singular values from the observed data to those from matrices with a given, known distribution such as the one described above. We next give a small example to show the different conclusions that can result if different definitions of  are used.

#### Example

**Figure 8:**
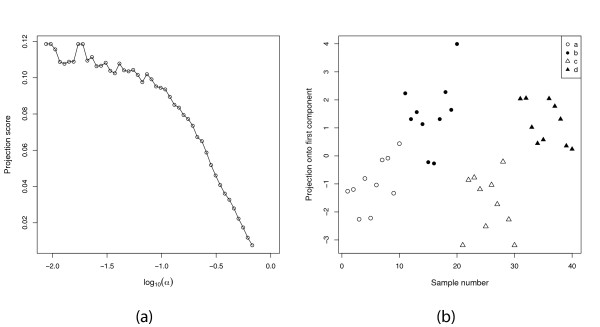
**The effect of the choice of null distribution**. (a) The projection score as a function of log_10_(*α*), where *α *is the *p*-value threshold obtained by applying a two-sided t-test for comparing two artificial sample groups, when  is defined by assuming all matrix elements are normally distributed. (b) The projection obtained by applying PCA to the most informative variable subset, containing 11 variables.

We define **X **∈ ℝ^1,000 × 40 ^by letting  for *i *= 1, ..., 1, 000; *j *= 1, ..., 40. We divide the samples into four consecutive groups *a*, *b*, *c*, *d *of 10 samples each, as in the response-related filtering above, and filter the variable set based on the absolute value of the *t*-statistic contrasting groups *a*∪*c *and *b*∪*d*, using *S *= {1}. If  is defined by assuming that each element of *ϕ_m_*(**X**) is drawn from a standard normal distribution, the most informative variable subset contains 11 variables. Here, even though we study a completely random matrix and an artificial grouping of the samples, we obtain a good separation between the groups. However, the projection score is not very high in this case (*τ *= 0.119). Figure 8 shows the projection score as a function of the significance threshold, and the optimal projection. As can be seen, just looking at the visualization in the right panel we might be led to believe that there is actually some non-random structure in the data. On the other hand, if we define  as we have done in the previous examples, by assuming independence among the samples and variables of the original matrix **X **and then filtering, no submatrix supports even *S *= {1}, and hence we get an indication that we are filtering a matrix without non-random relationships between the variables.

## Conclusions

In this paper, we have introduced and shown the usefulness of the projection score, a measure of the informativeness of a subset of a given variable set, based on the variance contained in the corresponding sample configuration obtained by PCA. The definition of the projection score is straightforward and the interpretation in terms of captured variance is intuitively appealing in a PCA visualization context. Moreover, the projection score allows a unified treatment of variable selection by filtering in the context of visualization, and we have shown that it indeed gives relevant results for three different filtering procedures, both for microarray data and for synthetic data. By filtering with respect to a specific factor, we obtain sparse principal components where all variables receiving a non-zero weight are indeed strongly related to the chosen factor. In this respect, the resulting components may be more easily interpretable than general sparse principal components, where the variables obtaining a non-zero weight can be related to many different factors. The optimal number of variables included in the extracted principal components often increases with |*S*| and in many cases small variable subsets do not even support large subsets *S*.

## Methods

### The projection score

#### Definition and basic properties

Let **X **= [**x**_1_, ..., **x***_N_*] ∈ ℝ^*p*×*N *^be a given matrix with rank *r*, containing *N *samples of *p *random variables. Principal Component Analysis (PCA) [1-4] reduces the dimensionality of the data by projecting the samples onto a few uncorrelated latent variables encoding as much as possible of the variance in the original data. Assuming that each variable is mean-centered across the samples, the empirical covariance matrix (scaled by *N *- 1) is given by **XX***^T^*. The covariance matrix is positive semi-definite with rank *r*, so by the spectral theorem we have a decomposition

Here **V **= [**v**_1_, ..., **v***_r_*] is a *p *× *r *matrix such that **V***^T^***V **= **I***_r_*, where **I***_r _*is the *r *× *r *identity matrix, and **Λ_X _**= *diag*(*λ*_1_(**X**), ..., *λ_r_*(**X**)) is the *r *× *r *diagonal matrix having the positive square root of the non-zero eigenvalues of **XX***^T ^*(that is, the singular values of **X**) in decreasing order along the diagonal.

The orthonormal columns of **V **contain the weights of the principal components, and the coordinates of the samples in this basis are given by **U **= **X***^T^***V**. We obtain a lower-dimensional sample configuration by selecting the columns of **U **with index in a specified subset *S *⊆ {1, ..., *r*}. The rows of this matrix then provide a representation of the samples in an |*S*|-dimensional space. In this paper the aim is to find particularly instructive such sample configurations for a given choice of *S*, by including only a subset of the original variables with non-zero weights in the principal components.

The first principal component is the linear combination of the original variables which has the largest variance, and the variance is given by . Similarly, the second principal component has the largest variance among linear combinations which are uncorrelated with the first component. Given a subset *S *⊆ {1, ..., *r *}, the fraction of the total variance encoded by the principal components with index in *S *is consequently

This interpretation implies that *α*_2 _can be used as a measure of the amount of information captured in the corresponding |*S*|-dimensional sample configuration. In some applications, it is fairly straight-forward to select a suitable subset *S*. The synthetic example above, where we filter the variables by their association to a response variable dividing the samples into two groups, is such a case, where we expect a one-dimensional signal and hence select *S *= {1}. In other applications, we may select *S *= {1, 2} or S = {1, 2, 3} in order to make visualization possible. It is also possible to try several different choices of *S *for the same data set, in order to possibly detect different patterns. A specific choice of *S *effectively indicates which part of the data is to be considered as the "signal" of interest, and the rest is in some sense considered irrelevant. For a given *S *the expected value of *α*_2 _depends heavily on the size and the underlying distribution of the matrix **X**. This should be taken into account in order to obtain a reasonable measure of the informativeness of **X**. We introduce the projection score as follows:

**Definition 1 **(Projection score). Let **X **∈ ℝ^*p*×*N *^be a matrix with rank *r*. For a given matrix probability distribution  and a subset *S *⊆ {1, ..., *r*} we define the projection score  by

where **Λ_X _**= (*λ*_1_(**X**), ..., *λ_r_*(**X**)) is the length-*r *vector containing the non-zero singular values of **X **in decreasing order.

While much work is going on in the field of random matrix theory and eigenvalue distributions for random matrices (see e.g. [36-38]), most results are asymptotic and only valid under certain distributional assumptions on the matrix elements. Since we do not expect such assumptions to hold true in general for submatrices obtained by variable selection procedures, it is often necessary to use randomization methods to estimate .

### The relationship between the projection score and the quality measure from the gene shaving algorithm

As described in the previous section, the projection score is a measure of the informativeness of a variable subset. Let **X **∈ ℝ^*k*×*N *^denote the submatrix corresponding to a certain variable subset. Letting *X_ij _*denote the value of the *i*'th variable in the subset for the *j*'th sample and assuming *S *= {1}, the informativeness measure used by the projection score is given by

where *β *= (*β*_1_, ..., *β_k_*) is the first principal component of **X**. The expression in the denominator is the variance of the variable subset, and the numerator is the variance of a weighted average of the variables in the subset, where each variable is weighted by the corresponding element of the first principal component. In [25,26], the authors use another quality measure of a variable subset, and apply it to subsets generated by a shaving procedure. The quality measure proposed in [25,26] is given by

where  denotes the overall mean value of **X**. To allow both positively and negatively correlated variables in the same cluster, the values are multiplied by the sign of the corresponding element of the first principal component of the variable subset before computing *R*^2^. This quality measure is similar to our informativeness measure, but all variables are weighted equally, by 1/*k*. Hence, in this case the Euclidean norm of the weight vector depends on *k*, while for the weight vector in the projection score we have . As noted in [26], the *R*^2 ^measure is biased towards small cluster sizes.

### How to use the projection score

In this section, we will outline how the projection score can be used to compare the informativeness of *M *different submatrices of a given matrix **X**. For a given collection of variable subsets *R_m_*, *m *= 1, ..., *M*, we define functions

for *m *= 1, ..., *M *by letting *ϕ_m_*(**X**) be a submatrix of **X **containing only the rows corresponding to the variables in *R_m_*. For filtering purposes, the number of variables to include in each submatrix (|*R_m_*|) can be determined by setting threshold levels on an underlying statistic in the observed matrix **X**. For example, one can successively include all variables with variances greater than 1%, 2%, . . . of the maximal variance among all variables. Note that this is only one example of how the variable subsets can be defined, and that there are many other possibilities. For example, we could successively include the 100, 200, . . . variables with the largest variances. Given a null distribution  for each *ϕ_m_*(**X**), we can calculate the projection score  for *m *= 1, ..., *M*. For a fixed *S*, a subset *R_m _*is then said to be *more informative *than a subset *R_n _*if

Note that the same *S *should be used for both subsets. In general, it is very difficult to compare projection scores obtained for different choices of *S*.

The null distribution , for matrices of dimension |*R_m_*| × *N*, can be defined in different ways. One particularly simple way is to assume that every matrix element is drawn independently from a given probability distribution, e.g.  for *i *= 1, ..., |*R_m_*| and *j *= 1, ..., *N*. Then, the optimal projection score is obtained for the submatrix whose singular values deviate most from what would be expected if all matrix elements were independent standard normally distributed variables. However, even if the original data set consists of independent normally distributed variables, this is not in general true after applying *ϕ_m_*. This means that even a submatrix obtained by filtering independent normally distributed variables may be far from the null distribution defined this way.

In the applications in this paper, we define  by permutation of the observed data, assuming that **X **consists of *N *independent samples of *p *independent variables. We then define the null distribution of each variable in *ϕ_m_*(**X**) by truncation of the corresponding distribution obtained from , with respect to the features of *ϕ_m_*. For real microarray data sets, which often contain a considerable amount of non-random variation, the variables are generally far from independent. Therefore we expect that the variance encoded by the leading principal components of the real data is larger than for the data generated under the null model, and the basic idea behind the projection score is to use the difference between them as an informativeness measure.

In practice, we generate *B *data matrices X*^b^, *b *= 1, ..., *B *from  by permuting the values in each row of **X **independently. For each X*^b ^we compute , and the expected value of (*α*_2_(**Λ_X_**, *S*))^1/2 ^under the probability distribution  is then estimated as

Similarly,  is estimated by repeated permutation of the values in each row of **X**, followed by application of *ϕ_m _*to the permuted matrix. Hence,

For each *b*, the variable subsets *R_m _*are re-defined in such a way that each *ϕ_m_*(X*^b^) contains the same number of variables as *ϕ_m_*(**X**). In our examples, we permute the data matrices *B *= 100 times.

When the number of variables is decreased by filtering, the true dimensionality of the resulting data set (that is, the number of non-trivial principal components) may change. We say that a submatrix *ϕ_m_*(**X**) *supports *a given *S *if the variance accounted for by each of the principal components of *ϕ_m_*(**X**) with index in *S *is large enough. More specifically, we estimate the distribution of  for each *k *∈ *S *under the probability distribution . If the estimated probability of obtaining a value of  at least as large as the observed value is less than 5% for all *k *∈ *S*, we say that the submatrix *ϕ_m_*(**X**) supports *S*. In practice, the null distribution of  is estimated from the singular values of the submatrices *ϕ_m_*(X*^b^). Permutation methods similar to this approach, comparing some function of the singular values between the observed and permuted data, have been used and validated in several studies to determine the number of components to retain in PCA [4,31,39].

### Generalizations of the projection score

In this section we will indicate some possible generalizations of the projection score defined above.

#### Generalized measure of information

We noted above that *α*_2_(Λ*_X_*, *S*) is a natural measure of the information contained in the principal components of **X **with index in *S*. More generally, we can use any ℓ^q^ norm (*q *≥ 1) instead of the ℓ^2 ^norm to measure information content, giving a measure of the explained fraction of the information content as

By replacing (*α*_2_(**Λ_X_**, *S*))^1/2 ^in the definition of the projection score with, for example, a function of the form

where *h *: ℝ → ℝ is an increasing function, we obtain a generalized projection score as

An alternative generalization is obtained by normalizing by taking the quotient of the observed and expected value of *g*(**Λ_X_**, *S*) instead of the difference, that is, defining the generalized projection score as

#### Supervised projection score

The projection score evaluates only the informativeness of the variable subsets with respect to visualization by PCA. We can make the variable subset selection (partially) supervised by incorporating a term quantifying the association of the variable subset with a response variable into the projection score. The term can be, for example, the classification ability of the variable subset (estimated by cross-validation) or the correlation between an aggregate of the variables and a quantitative response.

## Competing interests

The authors declare that they have no competing interests.

## Authors' contributions

MF contributed the idea of a projection score. MF and CS both contributed to the design of the projection score, to the implementation and numerical experiments, to the analysis and interpretation of the results and to the writing of the manuscript. Both authors read and approved the final manuscript.
